# Insights into Real Lignin Refining: Impacts of Multiple Ether Bonds on the Cracking of *β*-O-4 Linkages and Selectivity of Products

**DOI:** 10.3390/molecules31010133

**Published:** 2025-12-30

**Authors:** Yuancai Lv, Xuepeng Lin, Kai Yang, Yifan Liu, Xiaoxia Ye, Liang Song, Chunxiang Lin, Guifang Yang, Minghua Liu

**Affiliations:** 1 Fujian Provincial Engineering Research Center of Rural Waste Recycling Technology, College of Environment & Safety Engineering, Fuzhou University, Fuzhou 350116, China; yclv@fzu.edu.cn (Y.L.);; 2 Furen Group Co., Ltd., Fuzhou 350004, China; 3 Fujian Provincial Key Laboratory of Ecology-Toxicological Effects & Control for Emerging Contaminants, Putian University, Putian 351100, China; abc396550322@163.com

**Keywords:** lignin depolymerization, mixed ether bond, lignin structure, product hydrogenation

## Abstract

Depolymerizing lignin to produce high-value chemicals has garnered increasing attention. Given the complex structure of real lignin, the cracking efficiency of *β*-O-4 linkages and the selectivity of depolymerization products are significantly lower than those of lignin model compounds. Meanwhile, the relationship between the structure of lignin and the *β*-O-4 linkage cracking was ignored. In this work, to well address the issue, three real lignins (corncob lignin (CL), pinus massoniana lignin (PML), and eucalyptus lignin (EL)) were employed to discuss the impacts of special ether bonds in lignin on the *β*-O-4 linkage cracking in the no-additional-hydrogen catalytic system mediated by a CoNi_2_@BTC catalyst. The lignin depolymerization results showed that the ether bonding structure in the lignin significantly impacted the cracking of *β*-O-4 linkages and selectivity of the final products, resulting in a great difference among their intermediates. Notably, the methoxy groups in the real lignin greatly inhibited the further hydrogenation of phenolic compounds, resulting in the accumulation of abundant methoxy-substituted phenolic compounds and a low yield of cycloalkanes (12.37% to 14.06%). To deeply discuss the *β*-O-4 linkage cracking in the lignin depolymerization, degradation experiments with coexisting ether bond compounds were performed, and the activation energy was employed to quantitatively evaluate the impacts of other ether bonds on the *β*-O-4 linkage cracking. The results revealed that multiple ether bonds (*α*-O-4, 4-O-5, and methoxy group) significantly increased the activation energy (from 236% to 373%) of *β*-O-4 linkages, resulting in the evident decline in the *β*-O-4 model compound. In addition, the degradation of the methoxy-substituted *β*-O-4 model compound (GG) demonstrated that the methoxy-substituted aromatic ring products were resistant to further hydrogenation, resulting in the accumulation of methoxy-substituted aromatic ring products in the depolymerization of real lignin. All the findings will provide a novel perspective for the targeted high-value utilization of real lignin in chemical production.

## 1. Introduction

Lignin is the most abundant renewable aromatic biopolymer in nature. In this context, lignin has emerged as a critical feedstock for producing high-value chemicals [1,2]. Lignin constitutes 15–35% of lignocellulosic biomass. It is a complex three-dimensional network composed of phenolic units. These units are linked by various ether bonds, such as *β*-O-4, *α*-O-4, and 4-O-5 bonds, methoxy groups, and so on. Lignin provides a unique platform for the production of liquid fuels and aromatic compounds [3,4]. So far, ample studies reported that various lignins were able to produce various high-value chemicals (phenolic compounds, alkane, ester compounds, etc.) through catalytic depolymerization. For example, Shu et al. [5] used a palladium–carbon catalyst combined with metal chlorides to achieve the controlled depolymerization of lignin, resulting in the yield of guaiacol and phenol. According to Gao et al.’s findings [6], plenty of C_6_–C_10_ saturated alkane products were obtained through the hydrogenation and deoxygenation of real lignin oil by using a multi-metal oxoacid-supported single-atom Pt catalyst. Meanwhile, during the catalytic depolymerization of enzymatic and mild acid hydrolysis of lignin (EMAL) [7], the monomer phenol was yielded, as high as 47 wt%. Besides the phenolic compounds and alkane, small molecular ester compounds were also formed during the catalytic oxidative depolymerization of lignocellulosic biomass in ethanol mediated by a MoS_2_-MoC@NC800 catalyst [8]. All the reports demonstrated the feasibility of depolymerizing into fine chemicals.

During lignin depolymerization, there are two strategies to produce high-value chemicals, which involve producing phenolic compounds and cycloalkanes. For the former strategy, the core of lignin depolymerization was the selective cracking of various ether bonds, like *β*-O-4, *α*-O-4, 4-O-5, and methoxy groups, and preventing the further hydrogenation of phenolic compounds. For example, during the catalytic degradation of *β*-O-4, *α*-O-4, and 4-O-5 model compounds, the phenol yield efficiency achieved over 90% [9]. During the method of generating cycloalkanes, the core of lignin depolymerization involved both cracking of various ether bonds and further hydrogenation of phenolic compounds. During the catalytic process mediated by a single-atom Pt catalyst, plenty of C_6_–C_10_ saturated alkane products were formed [9]. However, in both methods of high-value utilization of lignin, there are great differences in the depolymerization efficiency and product distribution between lignin model compounds and real lignin under catalytic reactions [10,11,12,13,14]. For instance, during the catalytic system mediated by a bimetallic Ni-Mo-MOF catalyst [10], all the lignin model compounds were fully degraded. Instead, when the system was applied to real lignin, the lignin degradation efficiency was only 64.2%. Similarly, according to Zhu et al.’s findings [11], the lignin degradation efficiency (57.0%) was also much lower than that of lignin model compounds. The essence of this phenomenon fundamentally stemmed from their structural differences, particularly the coexistence of single ether bonds and multiple ether bonds. On the one hand, real lignin was a complex of various ether bonds, and its diverse inter-unit connections varied greatly due to their different sources, which would significantly impact the reactive activity of the *β*-O-4 linkage. On the other hand, during the depolymerization of real lignin, various by-products coexisted in the system, which meant the present by-products containing *α*-O-4 and 4-O-5 bonds would compete for the reactive sites on the catalyst surface with *β*-O-4 linkages, resulting in an increase in activation energy for bond cleavage [15].

In the catalytic depolymerization of real lignin, the diversity of ether bonds played a significant role in influencing the reaction pathways and product distributions [16]. Among lignin’s inter-unit linkages, the *β*-O-4 bond serves as a canonical model for studying substituent effects. Its phenolic ring bears methoxy (-OCH_3_) and hydroxyl (-OH) groups at variable positions, such as C2, C3, and C4, which can greatly alter the electronic density and steric environments [17]. In addition, these subtle structural differences dictate the interaction between catalysts and the substrate, leading to divergent product profiles, which range from phenols to cycloalkanes or oligomers [18]. For example, guaiacol-*β*-guaiacol, containing a C4-methoxy group (G-type lignin), and eugenol-*β*-eugenol, containing C3 and C5-dimethoxy groups (S-G-type lignin), exhibited different activities in hydrogenolysis reactions. During the degradation of G-type lignin and S-G-type lignin in the catalytic hydrogenolysis process mediated by a Ru/C catalyst, 92% of guaiacol-*β*-guaiacol was converted, which was much higher than that (68%) of eugenol-*β*-eugenol. The difference was due to the steric hindrance of the adjacent methoxy group [19]. In addition, the position of the methoxy substituent also influenced its sensitivity to ether bond cleavage. For example, in the 3-methoxy-*β*-O-4 model, the adjacent methoxy group hindered the sensitivity of the *β*-O-4 linkage, resulting in low degradation efficiency [20]. Therefore, understanding how substituents on the same ether bond type influence catalytic outcomes was also critical for developing tailored depolymerization strategies.

Based on previous reports, the complex structure of real lignin exhibited a significant impact on the distribution of its products. Some reports found that the product selectivity of the by-product was much lower than that of lignin model compounds; the authors thought that this was attributed to the complex ether bonds in the lignin, but they ignored the relationship between the by-product and the specific composition of ether bonds in the lignin and the impacts among various ether bonds during lignin depolymerization. To well address this issue, this work focused on the depolymerization of three real lignins with different ether bonds and deeply discussed the relationship between the selectivity of the final products and their structures in our previous catalytic system mediated by a CoNi_2_@BTC catalyst [21]. The detailed ether bonds in the real lignin structure were analyzed, and the lignins before and after catalytic depolymerization were well characterized by GPC, FTIR, and 2D HSQC NMR. In addition, the final products were also analyzed by GC-MS, and the selectivity of the products was also evaluated. Finally, to better clarify the mechanism among the various ether bonds on the selectivity of the products, coexistence systems with multiple ether bonds were designed, and their impacts on the cracking of *β*-O-4 linkages were evaluated. All the findings will provide a theoretical basis for the efficient conversion of lignin and the targeted regulation of desired products, as well as the further high-value utilization of real lignin.

## 2. Results and Discussion

### 2.1. Characteristics of Three Real Lignins

FTIR spectra clarify various structural components of the three different types of lignin (Figure S2). Regarding the specific attribution of lignin infrared spectra, refer to the classic literature on lignin structural characterization summarized in Table S1 [22]. From the figure, it can be seen that the spectral characteristics of the three lignins are similar, but there are differences in the intensity of certain absorption peaks. The bands at 1600, 1514, and 1421 cm^−1^ are assigned as aromatic skeletal vibrations of the lignin structure [23]. The broad peak centered at 3448 cm^−1^ is attributed to phenolic OH and aliphatic OH groups, and EL shows the highest intensity of this peak. It is worth noting that PML exhibits a strong guaiacyl (G) structural unit signal at 1270 cm^−1^; eucalyptus lignin shows a signal for the syringyl (S) structural unit at 1327 cm^−1^. Additionally, the spectrum of CL simultaneously shows signals for G, S, and p-hydroxyphenyl (H) structural units.

**Figure 1:**
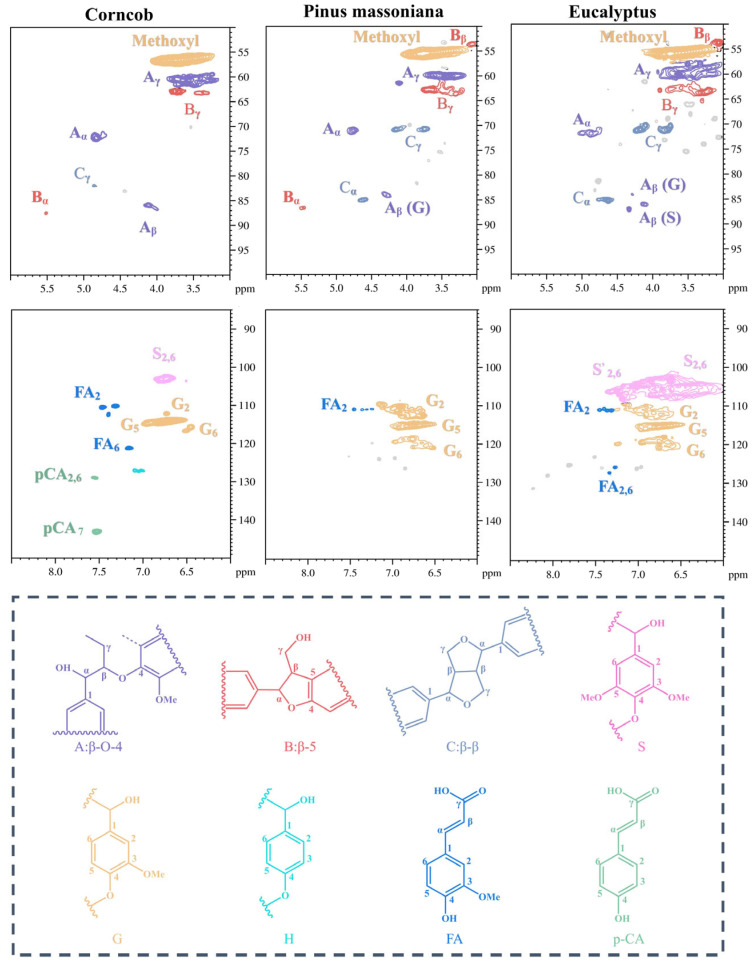
Side chain and aromatic regions in 2D HSQC NMR spectra of various lignins.

It should be noted that the origin of biomass could make lignin structurally complex. We then focused on revealing the lignins’ vastly different structures to gain deep insights into the depolymerization pathways of various lignins. Two-dimensional HSQC NMR was used to elucidate their initial structures in terms of S/G/H ratios and inter-unit linkages. The side chain region (δC/δH50−90/3−6), aromatic region (δC/δH100−150/6−8.5), and main substructures of the lignins are shown in Figure 1. In the aromatic region, the main cross-signals from the S, G, and H units were distinguished in the different lignins. The S units of EL exhibit strong characteristic signals in the NMR spectrum, further confirming the structural feature that S-type monomers dominate EL. The aromatic ring region of PML indicates that the lignin is primarily composed of G units, with a small amount of ferulic acid (FA) units. CL exhibits a richer variety of aromatic compounds. In its spectrum, in addition to the G units, signals for the S units and the H units, as well as signals for the p-coumaric acid (PCA) units, are detected.

In the side chain regions of the three types of lignins, cross-signals of methoxy groups and representative inter-unit bonds are observed. These bonds include *β*-O-4 (A), *β*-*β* (B), and *β*-5 (C). They are quantitatively analyzed through regional integration (Table 1). Comparative analysis of inter-unit bonds shows that the *β*-O-4 bond content in CL is the highest. In contrast, the *β*-O-4 bond content in eucalyptus lignin is the lowest. Notably, CL has the lowest methoxy content due to the higher content of G and H units. Conversely, EL contains a large amount of S units, resulting in the highest methoxy content.

Based on the FT-IR and 2D HSQC NMR analysis of the three types of lignin, PML can be classified as G-type softwood lignin. EL can be classified as GS-type hardwood lignin. CL can be classified as GSH-type grass lignin.

### 2.2. Catalytic Depolymerization of Real Lignin

**Figure 2:**
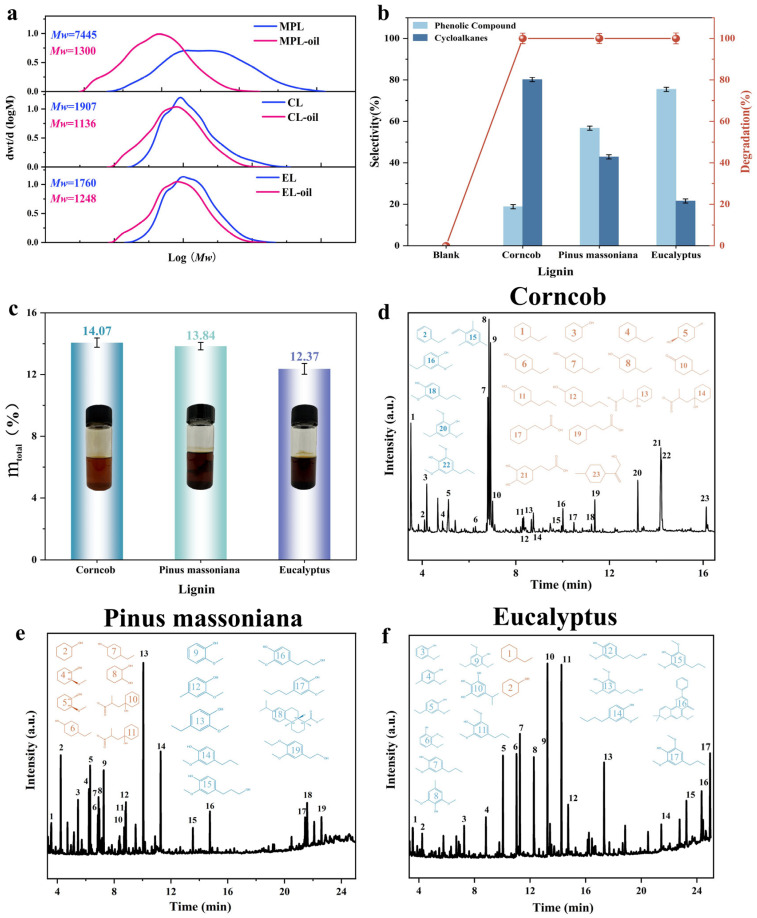
Depolymerization of various lignins: (**a**) molecular weight distributions, (**b**) conversion rate, and (**c**) total product yield. GCMS of bio-oil: (**d**–**f**).

The GPC curves indicate that the molecular weight distribution of the bio-oil produced after depolymerization of the three types has changed significantly compared to the original lignin (Figure 2a). The weight-average molecular weight (Mw) of the bio-oil from the depolymerized lignin of the three types was smaller than before depolymerization. This indicates that the three types of lignin were successfully depolymerized into small molecular substances under the catalytic system. Furthermore, only the PDI of EL increased from 1.82 to 2.05 after depolymerization. In contrast, the PDI of PML and CL decreased after depolymerization (Table S2). The differences in the molecular weight of depolymerization products are mainly attributed to the structural differences in lignin. The high proportion of S-type monomers in EL results in non-uniform cleavage during depolymerization. This leads to a broadening of the molecular weight distribution [24,25]. The weight changes during the lignin pyrolysis process are shown in Figure S3. The high S-type monomer content in EL is also a primary factor. It contributes to the greater weight loss during pyrolysis compared to CL and PML [26,27].

Research on the depolymerization products of CL, PML, and EL was conducted using GC-MS. As shown in Figure 2b, the depolymerization rates of the three types of lignin all reached 100%. Since no oxidation products were detected under the current conditions, it can be concluded that the oxidation process was very weak. Therefore, this result indicates that the catalyst has a strong depolymerization ability for lignin. We characterized the bio-oil from the depolymerization of three types of lignin using 2D HSQC NMR (Figure S4). And we performed semi-quantitative calculations on its structure (Table S3). From these analyses, we concluded that the catalysts achieved lignin depolymerization primarily by catalyzing *β*-O-4 bond cleavage in all three lignin types. However, there was a certain difference between the product yield and selectivity (Figure 2b–f).

According to the internal standard method and the calibration curves of products (Figure S5) for calculating product yield, the product yield of CL is the highest at 14.06%. PML follows with a yield of 13.84%. EL has the lowest yield at only 12.37%. This is because the *β*-O-4 content in the lignin linkage type of corncobs is the highest, while EL has the least. The catalyst mainly catalyzes the cleavage of *β*-O-4 to achieve depolymerization. In terms of product selectivity, the selectivity of phenolic substances in the depolymerization products of EL reaches 75.42%. The selectivity of naphthenic products is 21.59%. For PML, the selectivity of phenolic substances is 56.71%, and the selectivity of naphthenic products is 42.88%. For CL, the selectivity of phenolic substances is the lowest at 18.84%, while the selectivity of naphthenic products is the highest at 80.16% (Figure S6).

This difference is mainly attributed to the structural differences in the three types of lignin. EL belongs to S/G type lignin, with a higher proportion of S units and more methoxy substitutions on the aromatic rings. Previous studies have confirmed that the presence of methoxy groups enhances the stability of benzene rings [28]. This reduces the probability of hydrogenation reactions. As a result, highly selective phenolic products are produced. PML is mainly G-type lignin. It has a higher proportion of C-C bonds in the G unit. This results in a more complex structure and limited cleavage of C-O-C bonds. Under reaction conditions, some aromatic ring side chains undergo hydrogenation. This results in a distribution of phenolic and cycloalkane products. These products are close to each other in terms of their distribution. In contrast, CL primarily consists of H-type units. It has the lowest methoxy content in its structure. CL also has a high proportion of C-O-C bonds that are easily broken. Under catalytic hydrogenation conditions, phenolic rings are more readily reduced to naphthenes. This increases the selectivity for naphthene products and results in the lowest proportion of phenolic products.

The depolymerization results of the three different lignin structures confirm the previous experimental conclusion regarding ether bond cleavage. The results indicate that the coexistence of multiple ether linkages reduces the degradation efficiency of *β*-O-4 bonds. Additionally, methoxy-substituted aromatic products are difficult to hydrogenate.

**Figure 3:**
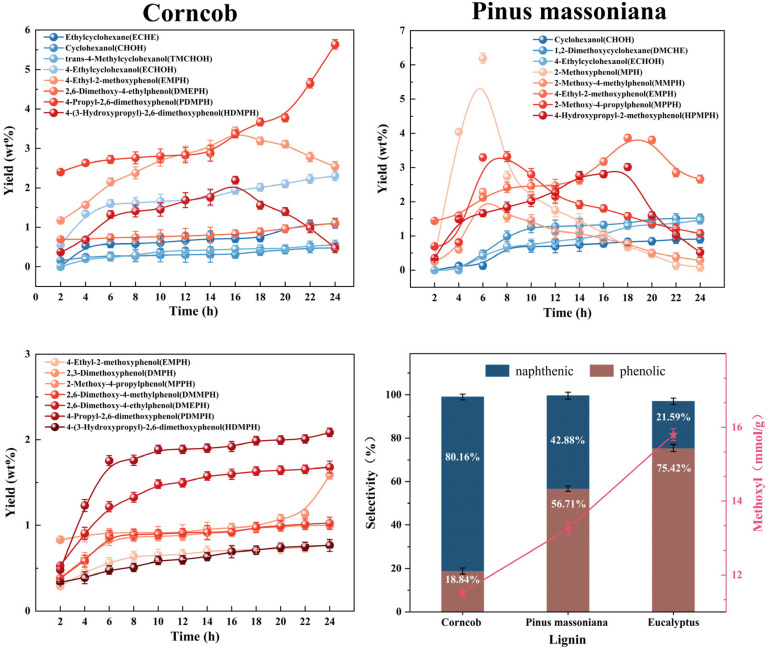
Changes in the depolymerization products of various lignins over time.

Furthermore, we analyzed the changes in the depolymerization products of the three types of lignin over time (Figure 3), selecting the seven or eight products with the highest content for explanation (Figures S7 and S8). First, we find that as the depolymerization time increases, the content of hydrogenated products among the three lignin depolymerization products gradually increases. This indicates that the catalyst has a relatively strong hydrogenation ability for the depolymerization products. Second, from the perspective of the types of lignin hydrogenation products, the products that can be hydrogenated almost all lack methoxy substitutions on the aromatic ring. This indirectly indicates that methoxy groups on the aromatic ring inhibit the hydrogenation of the benzene ring. This finding is consistent with the conclusions drawn earlier. Finally, we find that among the three lignin depolymerization products, the product with two methoxy groups on the aromatic ring rarely undergoes transformation as the depolymerization time increases. In contrast, the product with one methoxy group on the aromatic ring is more likely to undergo transformation with prolonged depolymerization time. This also illustrates the impact of the methoxy group on the stability of aromatic rings.

### 2.3. Influence Mechanism of Other Ether Bonds on the Catalytic Cracking of the β-O-4 Bond

**Figure 4:**
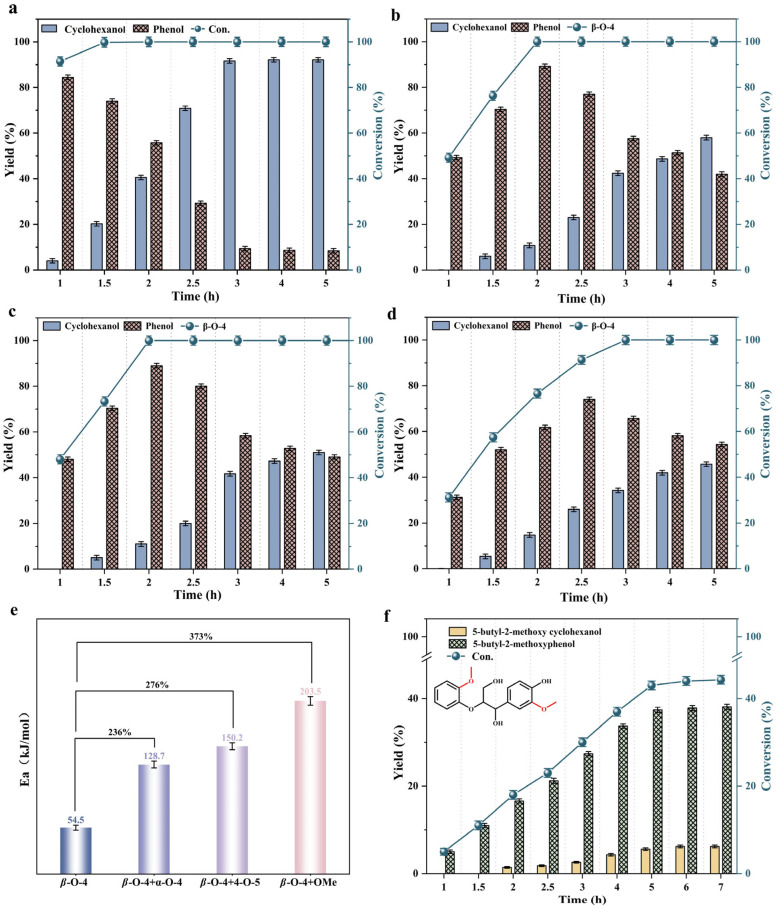
Mixed ether bonds within 5 h: (**a**) *β*-O-4; (**b**) *β*-O-4 + *α*-O-4; (**c**) *β*-O-4 + 4-O-5; (**d**) *β*-O-4 + methoxy group showing the conversion rate of *β*-O-4 and its product distribution; (**e**) activation energy of *β*-O-4 in mixed ether bonds; (**f**) depolymerization conversion rate and product distribution of methoxy-substituted *β*-O-4 dimer model compounds within 10 h. Reaction conditions: 0.05 mmol substrate, 1 g/L catalyst, 250 °C, and 20 mL isopropanol.

When *β*-O-4 was depolymerized alone at 250 °C, as shown in Figure 4a, *β*-O-4 was completely degraded within approximately 1.5 h. The product composition consisted of 76.04% phenol and 21.24% cyclohexanol—which is the hydrogenation product of phenol. This indicated that, before 1.5 h, the depolymerization process was primarily driven by the cleavage of ether bonds. It was not primarily driven by the hydrogenation of benzene-containing products. After 1.5 h, the concentration of phenol decreased while the concentration of cyclohexanol increased. At 5 h, the *β*-O-4 degradation products yielded only 8.38% phenol, while cyclohexanol accounted for 90.16%. This indicated that, after complete *β*-O-4 cleavage, the resulting phenol undergoes facile hydrogenation. This occurred in the presence of the catalyst. As a result, the depolymerization process shifted towards the predominance of arene ring hydrogenation products (Figure S9).

The depolymerization of *β*-O-4, with the incorporation of other ether linkages, is illustrated in Figure 4b–d. As illustrated in the diagram, the addition of various other ether linkages during the depolymerization of *β*-O-4 bonds resulted in a differential reduction in the catalyst’s efficiency for *β*-O-4 degradation. Among them, the methoxy group’s influence was the most significant. It resulted in a 50% reduction in the catalyst’s efficiency for *β*-O-4 bond cleavage. It is worth noting that as the depolymerization time increased, the distribution of *β*-O-4 depolymerization products increasingly approached the results obtained from their individual depolymerization (Figure S10). In other words, its hydrogenation effect on the depolymerization products was minimal.

Generally, the catalytic efficiency for *β*-O-4 depolymerization is closely related to the activation energy required [29]. The calculated activation energies for the *β*-O-4 linkage depolymerization in various mixed ether bond environments (Figure S11) are shown in Figure 4e. The presence of other ether bond types significantly increases the activation energy required for *β*-O-4 degradation. This increase is compared to the activation energy of *β*-O-4 alone, which is 54.5 kJ/mol. The increase in activation energy is attributed to the competitive depolymerization of other ether linkages and *β*-O-4, as well as electronic effects [30]. Competitive cleavage may occur during the depolymerization of other ether linkages and *β*-O-4, which elevates the energy barrier for *β*-O-4 depolymerization. Furthermore, the presence of other ether linkages may alter the electronic environment of the system, thereby influencing the reaction pathway of *β*-O-4. In the depolymerization of other ether linkages, the reactivity of *β*-O-4 may be altered by the electronic or steric effects of the oxygen atom. For instance, the depolymerization of other ether linkages may release intermediates or radicals. These intermediates or radicals alter the transition state stability of the *β*-O-4 depolymerization process. As a result, the energy required to overcome the corresponding reaction barrier increases [30].

As mentioned above, the presence of methoxy groups on different parent compounds of *β*-O-4 affects the efficiency of the catalyst in depolymerizing *β*-O-4 to some extent. We wanted to further investigate the impact of methoxy groups on *β*-O-4 depolymerization products. We selected a model compound of *β*-O-4 oligomers substituted with methoxy groups (GG) for the depolymerization experiment. The depolymerization results shown in Figure 4f indicate that the degradation efficiency of GG in the catalytic depolymerization system was not ideal. The degradation efficiency increased from 5% at 1 h to 44.3% at 10 h. Unlike the results with different parent forms of methoxy, the selectivity of hydrogenation products in the depolymerization products remained constant. As the depolymerization time was extended, it was always only 14.1%. This indicated that when a methoxy group was present on the benzene ring of the product, the catalyst’s hydrogenation effect on the product was significantly inhibited [31,32].

**Figure 5:**
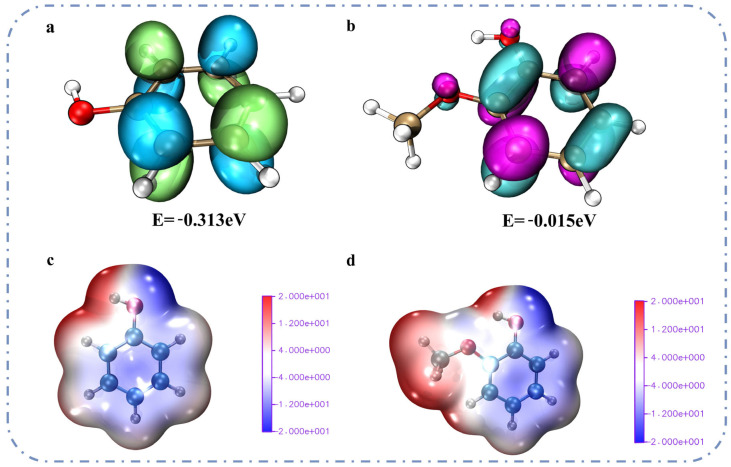
LUMO orbital energy: (**a**) phenol, (**b**) guaiacol; ESP: (**c**) phenol, (**d**) guaiacol.

In catalytic hydrogenation reactions, the hydrogenation of the benzene ring typically involves catalysts. These catalysts, such as metal surfaces, supply hydrogen atoms to the π system of the benzene ring. The LUMO orbital energy of the aromatic ring serves as a critical parameter. It determines the ability of the aromatic ring to accept hydrogen atoms [33,34]. Density functional theory (DFT) was used to calculate the LUMO orbital energies. The calculations were performed separately for guaiacol (with a methoxy substituent on the benzene ring) and for phenol (without any methoxy substitution on the benzene ring) (Figure 5a,b). From the perspective of molecular orbital theory, the LUMO of the benzene ring in aromatic hydrocarbons must overlap with the hydrogen atom orbitals. These hydrogen atom orbitals are provided by the catalyst during catalytic hydrogenation. A higher LUMO energy results in lower overlap efficiency and increased activation energy, making compounds like creosol more resistant to hydrogenation [35]. Conversely, phenol has a lower LUMO energy, facilitating electron acceptance and thus enabling easier hydrogenation [36,37]. We aimed to illustrate how methoxy substitution affects the benzene ring’s electron density. So, we carried out surface electrostatic potential calculations. These calculations were performed for guaiacol and phenol using DFT (Figure 5c,d). In guaiacol, the blue region on the benzene ring is more intense than in phenol, indicating a higher electron density on the benzene ring in guaiacol compared to phenol. This is due to the electron-donating resonance (+M) effect of the methoxy group (-OCH_3_). This effect significantly outweighs its electron-withdrawing inductive effect (-I). As a result, electrons are injected into the π system of the benzene ring [38]. The electron-donating effect of the hydroxyl group in phenol is relatively weak. As a result, the overall electron density on the benzene ring in guaiacol is higher compared to phenol [39]. According to molecular orbital theory, an increase in electron density raises the energy of π orbitals. This, in turn, leads to an elevation in LUMO energy [40]. This result is consistent with our previous DFT calculations of LUMO energy.

## 3. Experimental Section

### 3.1. Chemicals and Materials

In this work, the catalyst (CoNi_2_@BTC) (Figure S1) used was reported in previous work [21]. The lignin model compounds, including 2-phenylethyl phenyl ether(*β*-O-4), benzyl phenyl ether (*α*-O-4), diphenyl ether (4-O-5), anisole, m-Dimethoxy benzene, guaiacylglycerol-*β*-guaiacyl ether (GG), were all purchased from Aladdin Industrial Company (Shanghai, China). Corncob lignin, pinus massoniana lignin, and eucalyptus lignin were sourced from Jinan Koste Experimental Equipment Co., Ltd. (Jinan, China). Isopropanol, hydriodic acid, and sodium citrate dihydrate were provided by China Pharmaceutical Group Co. (Shanghai, China). All commercially purchased chemicals were used without further purification.

### 3.2. Decomposition of Lignin Model Compounds and Real Lignin Under an Air Atmosphere

The decomposition experiments were performed in a parapolybenzene autoclave reactor. For the degradation of the lignin model compounds (PPE, BPE, DPE, and GG), 0.05 mmol of the lignin model compounds, 1 g/L of the catalyst, and 20 mL of isopropanol were, in turn, added into the reactor. After sonication for 10 min, the hydrothermal reactor was sealed and then kept in a blast drying oven for the desired time. Once the reactor cooled down, the diluted organic products were evaluated. They were assessed both qualitatively and quantitatively. A Shimadzu quadrupole gas chromatography coupled with a mass spectrometer (GCMS-QP202NX, Shimadzu, Kyoto, Japan) was used for the evaluation.

For real lignin depolymerization, 1 g of lignin, 500 mg of the catalyst, and 20 mL of isopropanol were added to the reactor, and then, the reactor was kept at 250 °C. After the reaction, the lignin depolymerization products were analyzed with GCMS-QP202 NX. The unit of yield was mg/g lignin. All experiments were independently repeated three times, and the data were presented as the mean ± standard deviation.

### 3.3. Characterization of Lignins and Depolymerized Products

Structural characterizations of the lignins were carried out by Fourier transform infrared spectroscopy (FTIR, Nicolet iS20, Thermo Fisher Scientific, Waltham, MA, USA). The molecular weight and distribution of the lignins were characterized by gel permeation chromatography (GPC), using the Agilent PL-GPC220 system. According to the method of Li et al. [41], the methoxy content in the lignin samples was determined using the HS-GC method. Two-dimensional heteronuclear single quantum correlation nuclear magnetic resonance (2D HSQC NMR) analysis was carried out on a Bruker AVIII 500 MHz spectrometer (Bruker, Berlin, Germany). MestReNove 16 software was used to process all NMR spectra. For the HSQC NMR experiment, approximately 80 mg of lignin was dissolved in 0.6 mL of DMSO-d_6_. A semi-quantitative HSQC analysis was performed using volume integrals of correlation peaks. The results were expressed as the percentage of per 100 aromatic rings (Ar) [42,43].

$$\begin{array}{c} \mathrm{Total}\;\mathrm{aromatics}\;\left(100\;C_{9}\;\mathrm{units}\right)=\left(S_{2,6}+S_{2,6}^{\prime }+H_{2,6}\right)/2+S_{\mathrm{condensed}}+\left(G_{2}+G_{\mathrm{condensed}}\right)\\ S\%=\left[\left(S_{2,6}+S_{2,6}^{\prime }\right)/2+S_{\mathrm{condensed}}\right]/\mathrm{Total}\;\mathrm{aromatics}\times 100\%\\ G\%=\left(G_{2}+G_{\mathrm{condensed}}\right)/\mathrm{Total}\;\mathrm{aromatics}\times 100\%\\ H\%=\left(H_{2,6/2}\right)/\mathrm{Total}\;\mathrm{aromatics}\times 100\%\\ S/G\;\mathrm{ratio}=\left[\left(S_{2,6}+S_{2,6}^{\prime }\right)/2+S_{\mathrm{condensed}}\right]/\left(G_{2}+G_{\mathrm{condensed}}\right) \end{array}$$

Using the formula, the total aromatic content was calculated. It was based on the volume integration of the signals corresponding to syringyl units (S_2,6_, S’_2,6_, and S_condensed_), guaiacyl units (G_2_ and G_condensed_), and p-hydroxyphenyl units (H_2,6_). The total aromatic content was used as the internal standard (100 C_9_ units). The total number of linkages per 100 C_9_ units was based on the signal of the α position of the linkages. The total number of linkages was calculated with the following formulas:

$$\begin{array}{c} \beta \text{-}O\text{-}4\;\mathrm{linkages}=(\beta \text{-}O\text{-}4_{\alpha })/\mathrm{Total}\;\mathrm{aromatics}\times 100\%\\ \beta \text{-}\beta \;\mathrm{linkages}=(\beta \text{-}\beta_{\alpha })/\mathrm{Total}\;\mathrm{aromatics}\times 100\%\\ \beta \text{-}5\;\mathrm{linkages}=(\beta \text{-}5_{\alpha })/\mathrm{Total}\;\mathrm{aromatics}\times 100\% \end{array}$$

The conversion rate, selectivity, and yield of the model compounds and lignin depolymerization products were analyzed using Shimadzu QP2020NX gas chromatography–mass spectrometry (GC-MS, Shimadzu, Kyoto, Japan).

The lignin and model compound conversion rates and selectivity were calculated as follows:

$$\begin{array}{c} \mathrm{Conversion}\;\mathrm{rate}=\frac{\;\mathrm{number}\;\mathrm{of}\;\mathrm{moles}\;\mathrm{of}\;\mathrm{reacted}\;\mathrm{substrate}\;}{\;\mathrm{total}\;\mathrm{molar}\;\mathrm{injection}\;\mathrm{of}\;\mathrm{substrate}\;}\times 100\%\\ \mathrm{Selectivity}\;\left(X\right)=\frac{\;\mathrm{number}\;\mathrm{of}\;\mathrm{moles}\;\mathrm{of}\;X}{\;\mathrm{total}\;\mathrm{molar}\;\mathrm{injection}\;\mathrm{of}\;\mathrm{substrate}\;} \end{array}$$

Lignin product yield was calculated as follows:

$$m_{i}=\frac{A_{i}}{A_{IS}}\times m_{IS}$$

$m_{i}$: the mass of the target compound (mg);

$A_{i}$: the peak area of the target compound;

$A_{IS}$: the peak area of the internal standard (acetophenone);

$m_{IS}$: the mass of the internal standard (mg).

$$m_{S}=\frac{m_{i}}{\sum m_{i}}\times 100\%$$

$m_{S}$: the selectivity of a certain type of product.

### 3.4. Activation Energy Calculation

The calculation formula of activation energy is as follows:

$$\alpha =1-e^{-kt}$$

*α*: the depolymerization rate;

*k*: the reaction rate constant;

*t*: the reaction time (h).

$$k=A\cdot e^{-\frac{E_{a}}{RT}}$$

*A*: the pre-exponential factor (s^−1^);

*E_*α*_*: the activation energy (J/mol);

*R*: the ideal gas constant (8.314 J/mol/K);

*T*: the absolute temperature (K);

All experiments were independently repeated three times, and the data were presented as the mean ± standard deviation.

## 4. Conclusions

In the CoNi_2_@BTC/isopropanol transfer hydrogenation decomposition system, the oxidation pathway should be greatly inhibited. The difference in the structures of three real lignins (corncob lignin (CL), pinus massoniana lignin (PML), and eucalyptus lignin (EL)) led to a great difference among their intermediates in the obtained bio-oil. Notably, the methoxy groups in the real lignin greatly inhibited the further hydrogenation of phenolic compounds, resulting in the accumulation of abundant methoxy-substituted phenolic compounds and a low yield of cycloalkanes (12.37% to 14.06%). The great difference in the bio-oil was attributed to the multiple ether bonds in the real lignin, which played an important role in the cracking of *β*-O-4 linkages and the further hydrogenation process of the phenolic products. During the lignin depolymerization, the co-existing multiple ether bonds (*α*-O-4, 4-O-5, and methoxy group) significantly increased the activation energy (from 236% to 373%) of *β*-O-4 linkages, resulting in the evident decline in *β*-O-4 linkage cracking. In addition, the methoxy-substituted aromatic ring products were resistant to further hydrogenation, resulting in the accumulation of methoxy-substituted aromatic ring products in the depolymerization of real lignin. All the findings will provide a novel perspective for the targeted high-value utilization of real lignin in chemical production.

## Figures and Tables

**Table 1:** Quantification of various lignin inter-unit linkages (%).

Lignin	S/G/H ^a^	S/G	*β*-O-4 ^b^	*β*-*β* ^b^	*β*-5 ^b^	Methoxyl (mmol/g)
CL	35/49/16	0.71	89.8	trace	10.2	11.5
PML	-/99/-	-	72.9	17.0	20.1	13.3
EL	76/24/-	3.22	65.3	34.7	trace	15.8

^a^ Results are expressed per 100 Ar based on quantitative 2D-HSQC spectra. ^b^ The amount of the specific functional group is expressed as the percentage of *β*-O-4 + *β*-*β* + *β*-5.

## Data Availability

Data are contained within the article and Supplementary Materials.

## Supplementary Materials

The following supporting information can be downloaded at: https://www.mdpi.com/article/10.3390/molecules31010133/s1, Figure S1: (a) XRD patterns; (b) Nitrogen adsorption-desorption isotherms; (c) Ni 2p spectra; (d) Raman spectra; (e) Py-Ir spectra; (f) NH_3_-TPD of CoNi_x_@BTC; Figure S2: FTIR spectra of various lignins; Figure S3: (a) TGA and (b) DTG spectra of various lignins; Figure S4: Side-chain and aromatic region in 2D HSQC NMR spectra of various bio-oil; Figure S5: The calibration curves of some of the main products obtained through GCMS; Figure S6: The depolymerization of various lignins by the recycled catalyst; Figure S7: The top several degradation products of various lignins change over time; Figure S8: GC-MS mass spectra of the degradation of various lignins; Figure S9: The depolymerization mechanism of the main ether bonds in lignin; Figure S10: Mixed ether bonds within 10 h: (a) *β*-O-4 + *α*-O-4; (b) *β*-O-4 + 4-O-5; (c) *β*-O-4 + methoxy group; (d) *β*-O-4 + *α*-O-4 + 4-O-5 + methoxy group showing the conversion rate of β-O-4 and its product distribution. Reaction conditions: 0.05 mmol substrate, 1g/L catalyst, 250 °C, and 20 mL isopropanol; Figure S11: Activation energy of *β*-O-4 in mixed ether bonds: (a) *β*-O-4; (b) *β*-O-4 + *α*-O-4; (c) *β*-O-4 + 4-O-5; (d) *β*-O-4 + OMe; Table S1: FTIR spectral analysis; Table S2: The molecular weight distributions; Table S3: Quantification of various lignin bio-oil interunit linkages (%); Table S4: Assignment of ^13^C-^1^H cross signals in HSQC.
