# Supplementary material for: Insights into Real Lignin Refining: Impacts of Multiple Ether Bonds on the Cracking of β-O-4 Linkages and Selectivity of Products
Source: Molecules. 2025 Dec 30;31(1):133. doi: 10.3390/molecules31010133 (PMC12786805; doi:10.3390/molecules31010133)
Supplement: Supplementary file 1 [file molecules-31-00133-s001.zip › molecules-4020262-supplementary.pdf]

Supporting information for
Insights to real lignin refining: impacts of multiple ether bonds on the cracking of β-O-4 linkages and selectivity of products

Yuancai Lv[a,1], Xuepeng Lin[a,2], Kai Yang[b,3], Yifan Liu[a,4], Xiaoxia Ye[a,5], Liang Song[a,6] Chunxiang Lin[a,*], Guifang Yang[c, 7], Minghua Liu[a,c **]

[a] Fujian Provincial Engineering Research Center of Rural Waste Recycling Technology, College of Environment & Safety Engineering, Fuzhou University, Fuzhou 350116, China.

[b] Furen Group Co., Ltd, Fuzhou 350004, China.

[c] Fujian Provincial Key Laboratory of Ecology-Toxicological Effects & Control for Emerging Contaminants, Putian University, Putian, 351100, China

*Corresponding author. Email: abc396550322@163.com; Fujian Provincial Key Laboratory of Ecology-Toxicological Effects & Control for Emerging Contaminants, Putian University, 1133 Xueyuan Road, Chengxiang District, Putian, Fujian Province 351100, China.

**Corresponding author. Email: mhliu2000@fzu.edu.cn; College of Environmental and Biological Engineering, Putian University, 1133 Xueyuan Road, Chengxiang District, Putian, Fujian, 351100, China.

[1] Email: yclv@fzu.edu.cn, [2] Email: 230620039@fzu.edu.cn, [3]Email: kaiy0728@163.com, [4] Email: yfanym@fzu.edu.cn, [5] Email: yexiaoxia@fzu.edu.cn, [6] Email: songliang@fzu.edu.cn, [7] Email: abc396550322@163.com

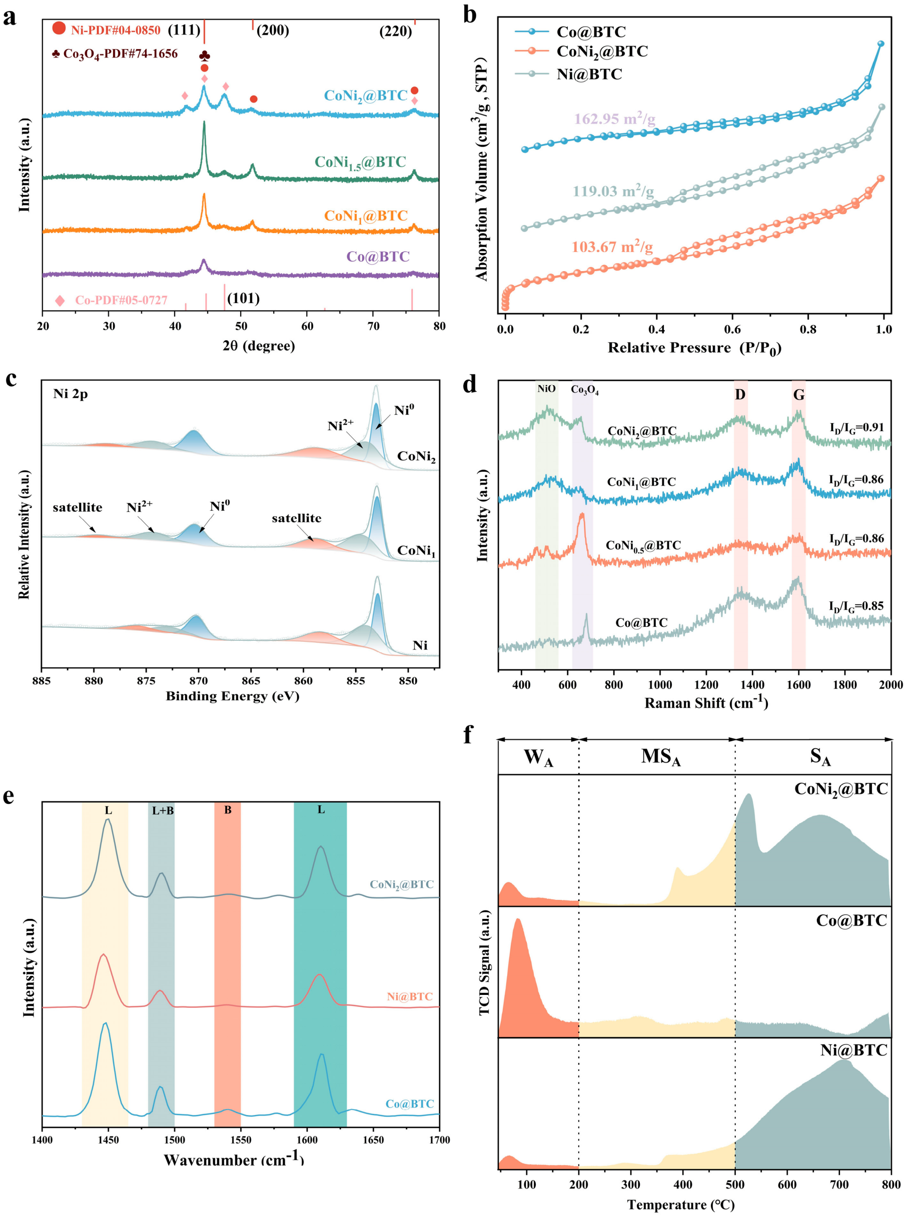

Figure S1 (a) XRD patterns; (b) Nitrogen adsorption-desorption isotherms; (c) Ni 2p spectra; (d) Raman spectra; (e) Py-Ir spectra; (f) NH_3-TPD of $CoNi_x$@BTC.

As can be seen from Figures S1a, S1c, and S1d, the crystal structure of the $CoNi_2$@BTC catalyst consists of gold Co, metallic Ni, and a small amount of Co_3O_4. From Figure S1b, it can be observed that the $CoNi_2$@BTC catalyst has a rich microporous structure and a relatively small specific surface area. From Figures S1e and S1f, it can be seen that the $CoNi_2$@BTC catalyst has abundant B and L acid sites, which also endows the catalyst with high performance.

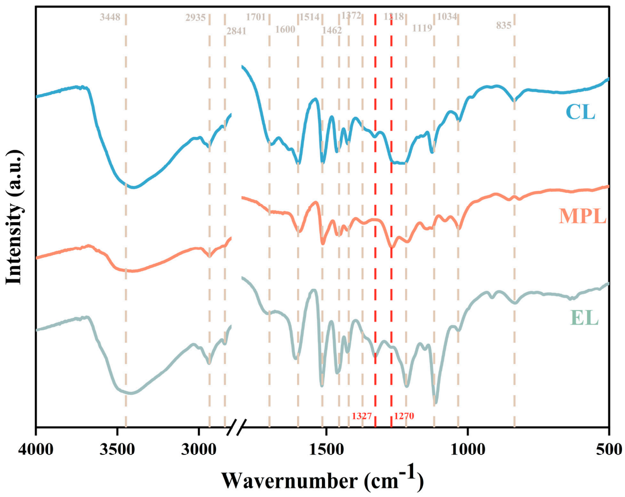

Figure S2 FTIR spectra of various lignins.

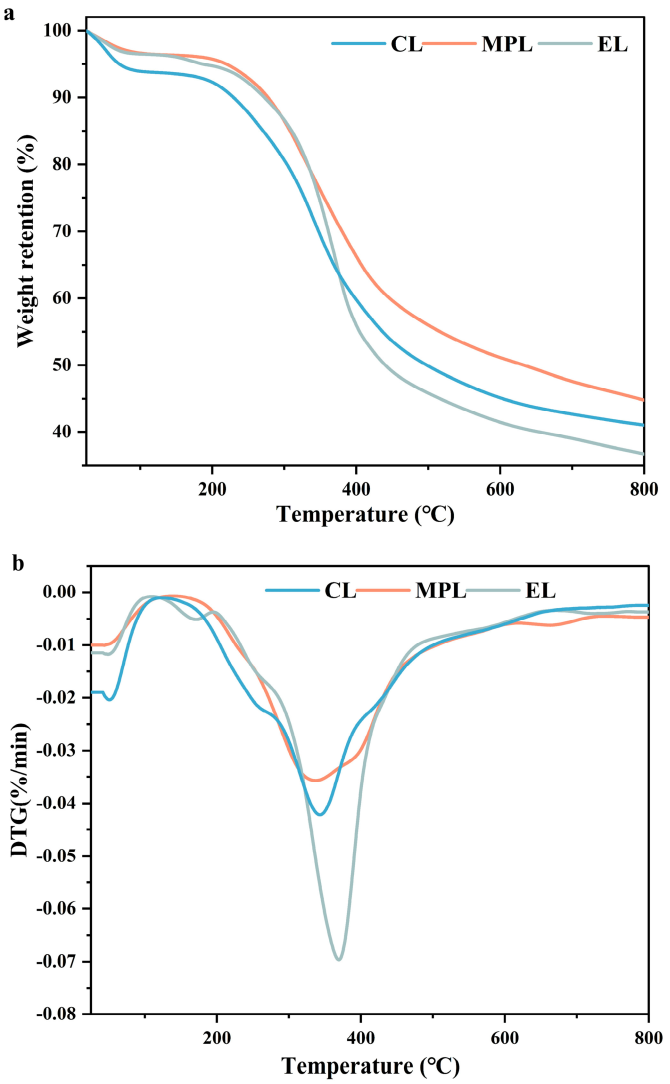

Figure S3 (a) TGA and (b) DTG spectra of various lignins.

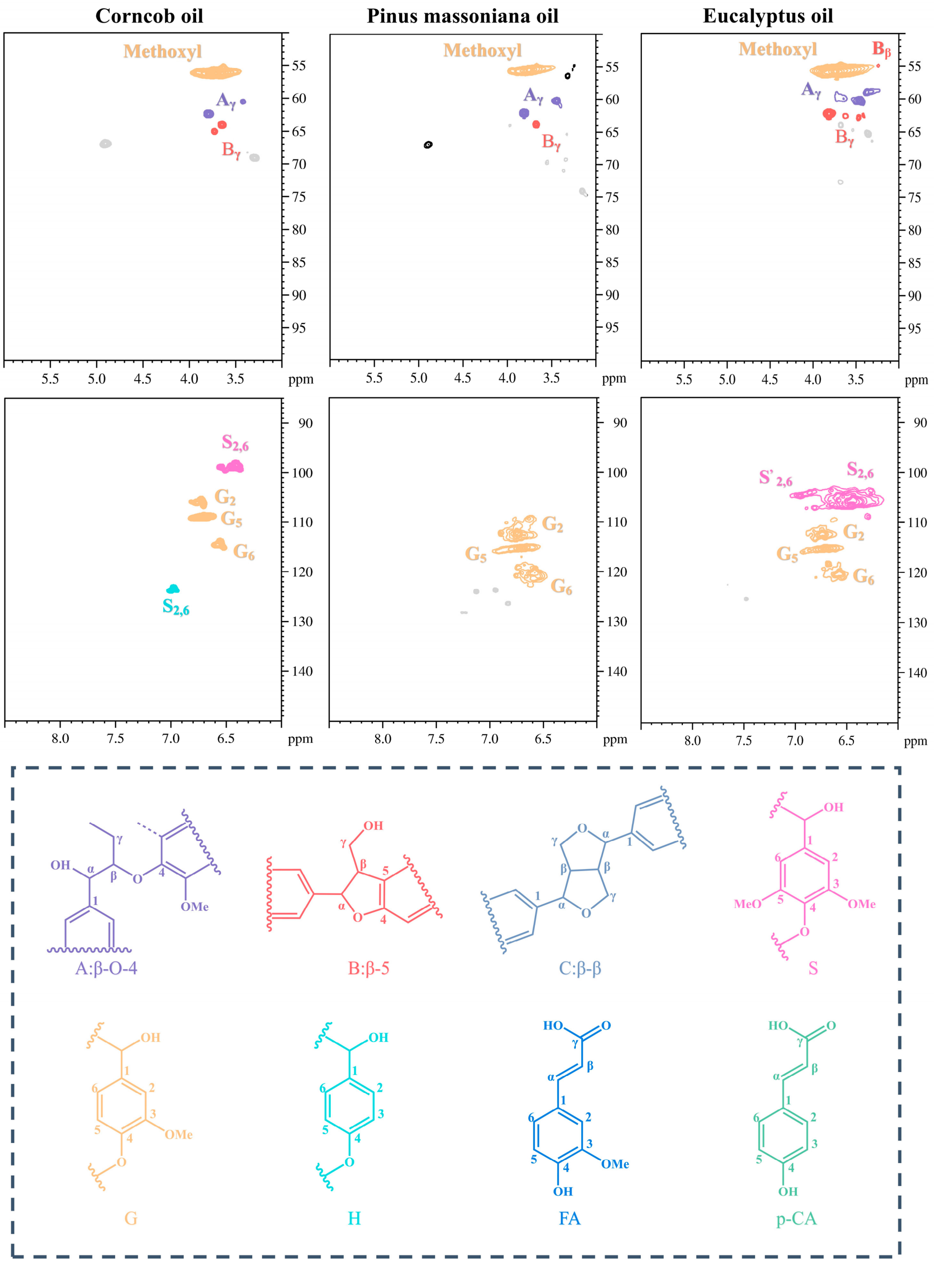

Figure S4 Side-chain and aromatic region in 2D HSQC NMR spectra of various bio-oil.

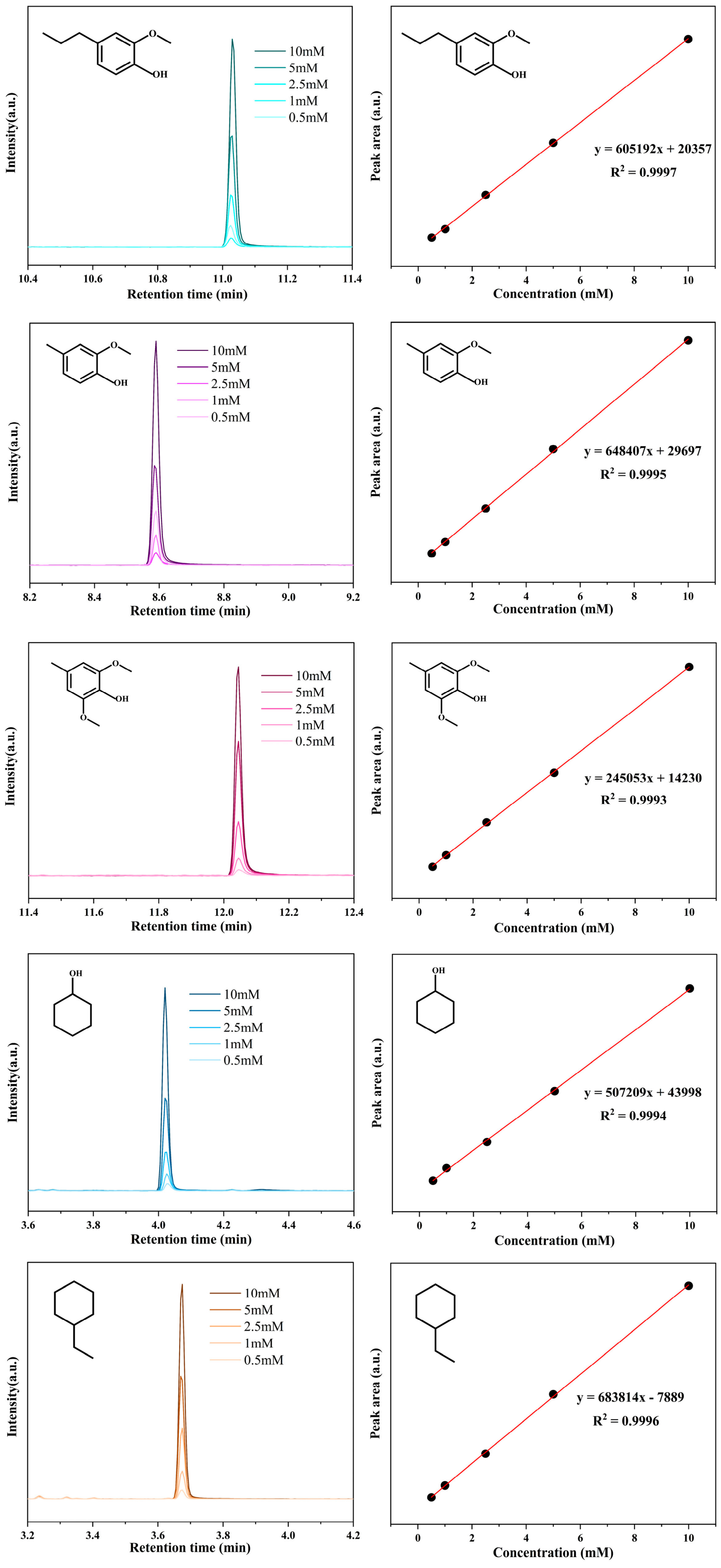

Figure S5 The calibration curves of some of the main products obtained through GCMS.

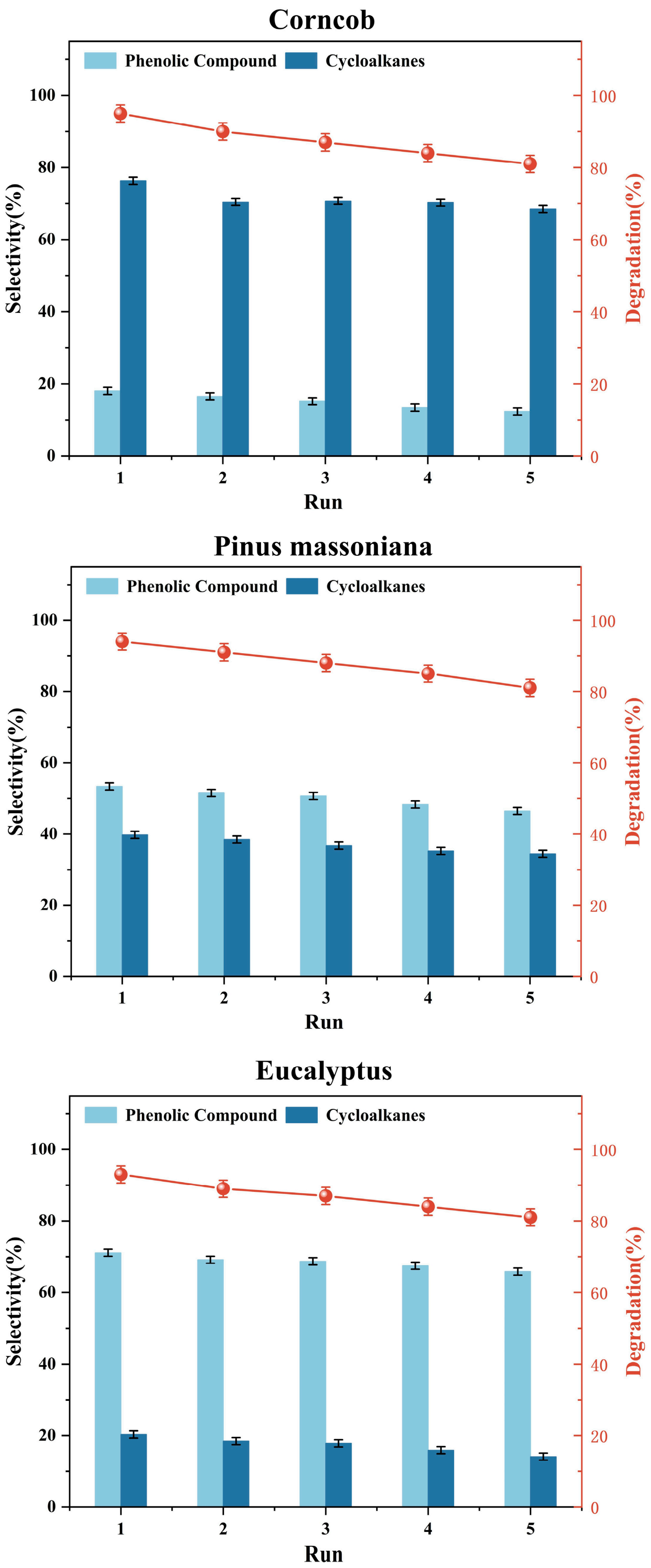

Figure S6 The depolymerization of various lignins by the recycled catalyst.

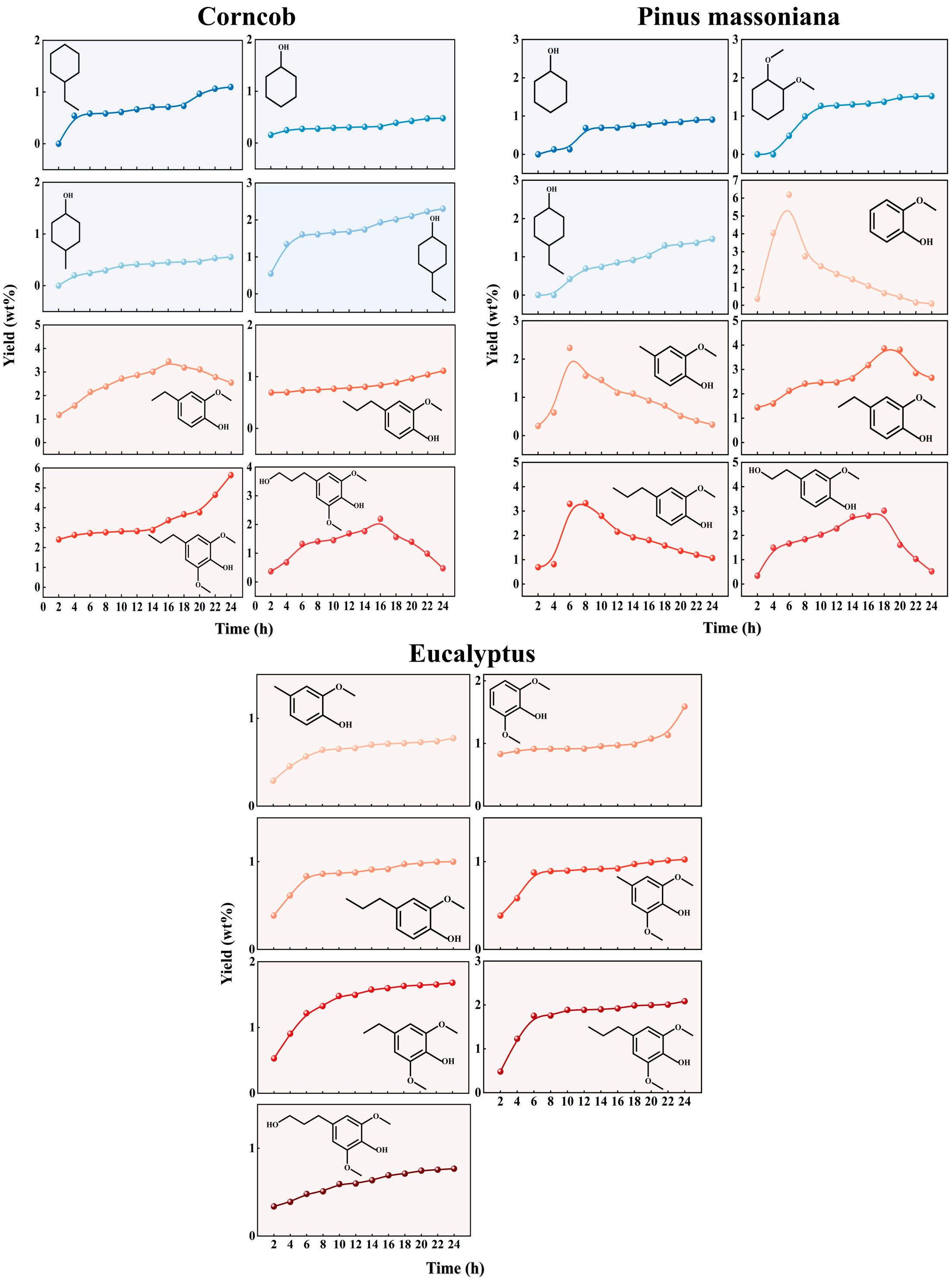

Figure S7 The top several degradation products of various lignins change over time.

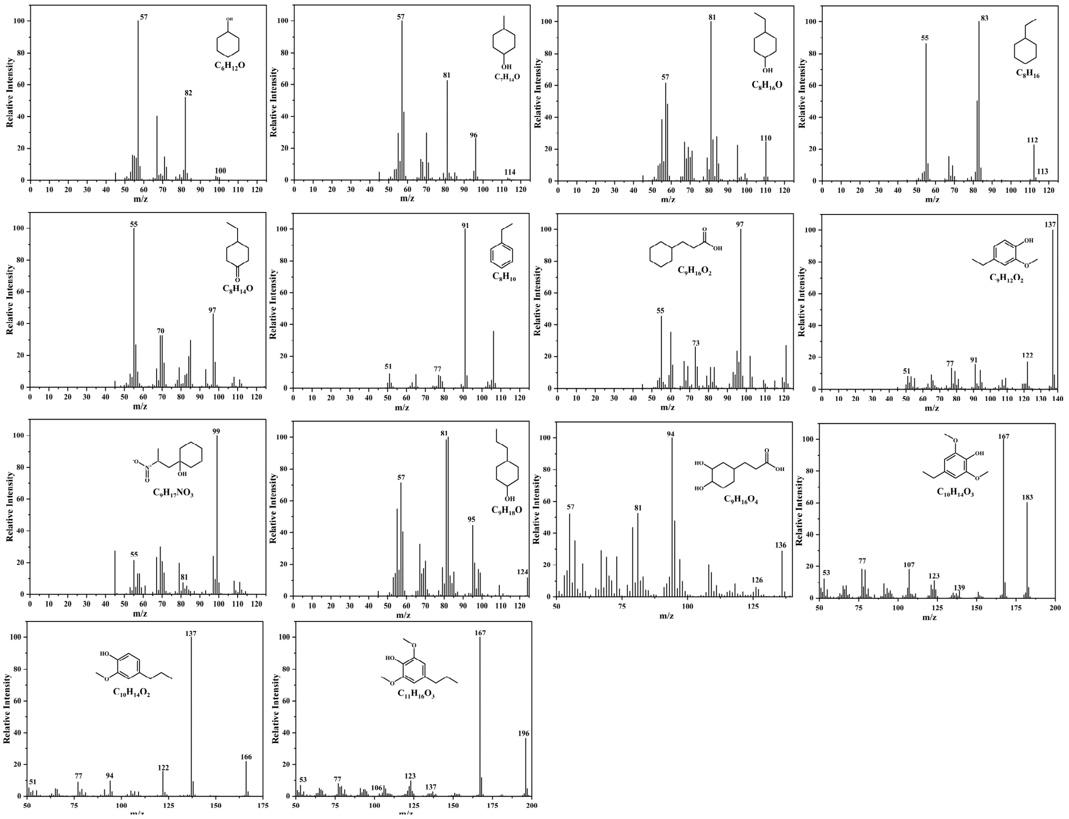

Figure S8 GC-MS mass spectra of the degradation of various lignins.

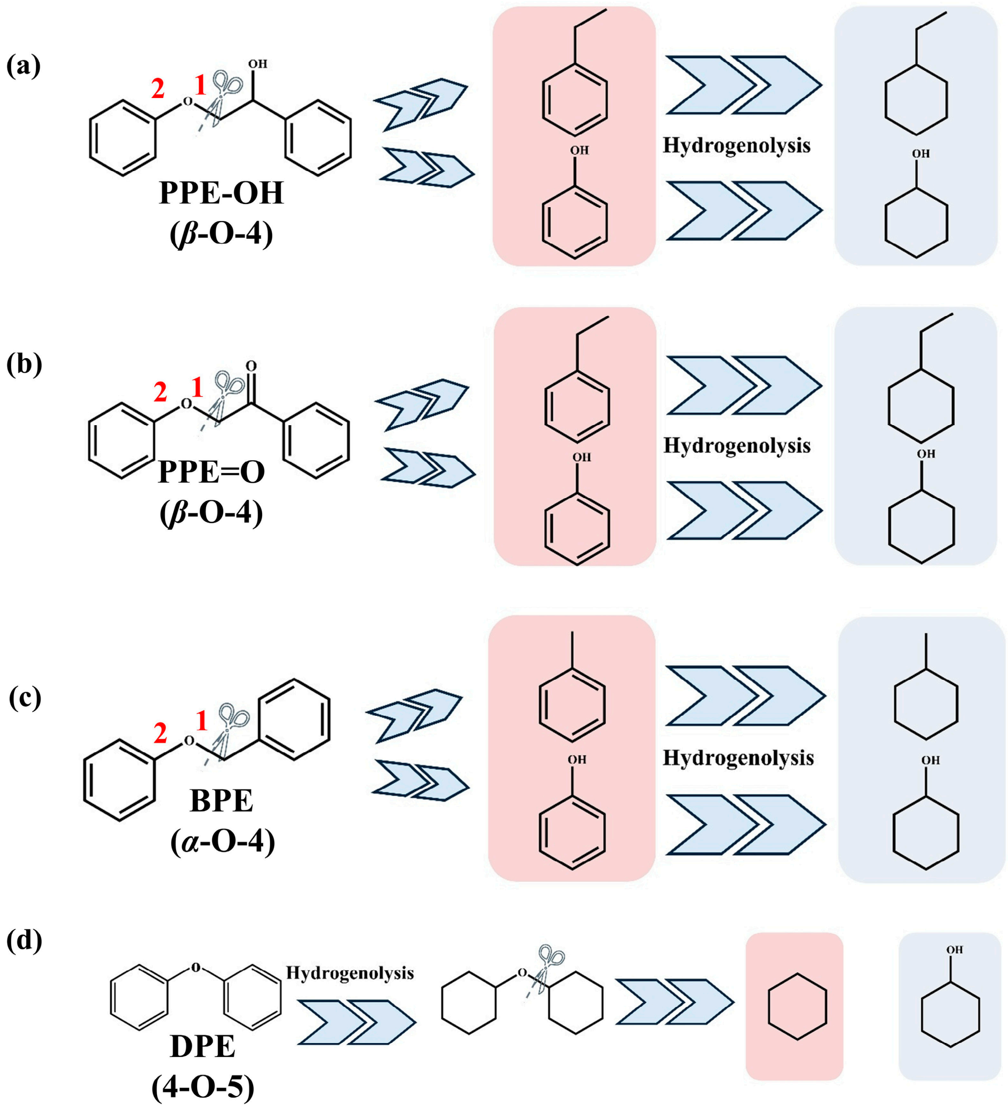

Figure S9 The depolymerization mechanism of the main ether bonds in lignin.

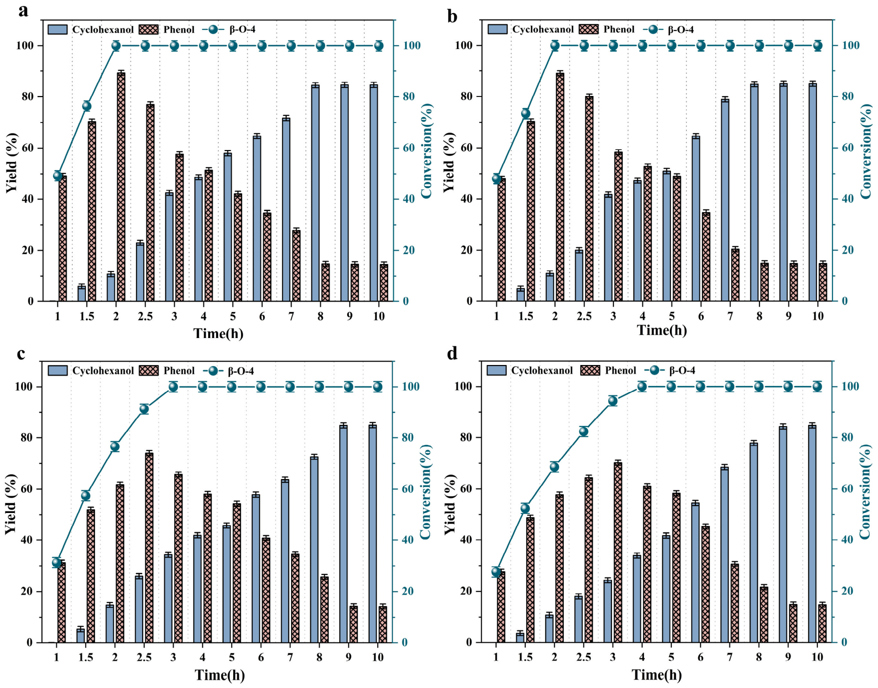

Figure S10 Mixed ether bonds within 10 hours: (a) β-O-4 + α-O-4; (b) β-O-4 + 4-O-5; (c) β-O-4 + methoxy group; (d) β-O-4+ α-O-4+ 4-O-5 + methoxy group showing the conversion rate of β-O-4 and its product distribution. Reaction conditions: 0.05 mmol substrate, 1g/L catalyst, 250℃, and 20 mL isopropanol.

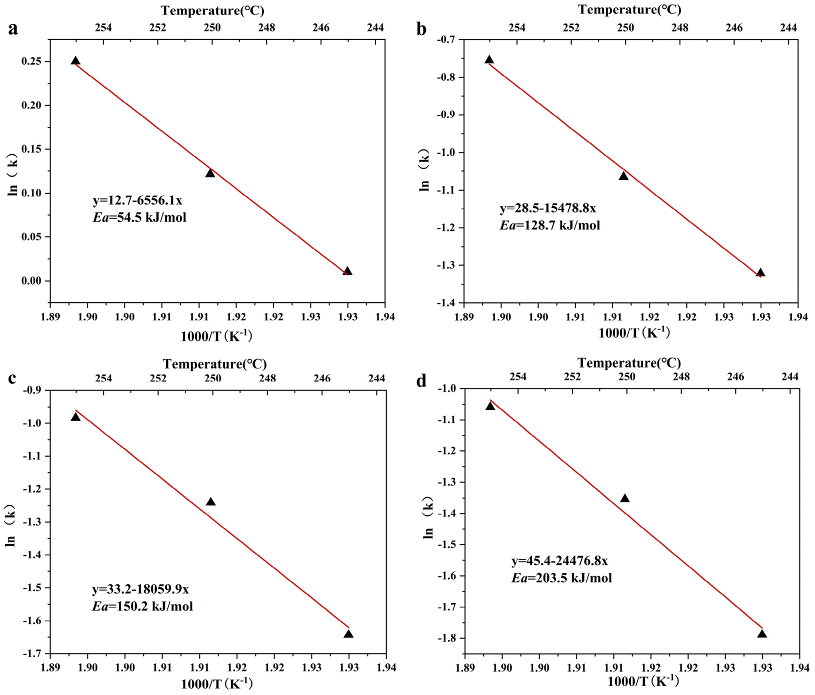

Figure S11 Activation energy of β-O-4 in mixed ether bonds: (a) β-O-4; (b) β-O-4 + α-O-4; (c) β-O-4 + 4-O-5; (d) β-O-4 + OMe.

Table S1. FTIR spectral analysis.

Number	Wave number (cm^{-1})	Absorption peak attribution
1	3448	-OH telescopic vibration.
2	2935	$-OHC_3$ telescopic vibration.
3	1701	non-conjugated ketones, carboxyl groups, and ester carbonyl (C=O) stretching vibrations.
4	1600	aromatic hydrocarbon framework and C=O stretching vibrational modes.
5	1514	vibrational modes of aromatic hydrocarbon frameworks (G>S).
6	1455	C-H bond variation.
7	1421	vibrational modes of the benzene ring skeleton.
8	1372	stretching vibrational mode of the C-H bond on the side chain.
9	1327	fundamental structural unit of the S-shaped configuration.
10	1270	fundamental structural unit of the G-shaped configuration.
11	1218	C–C, C–O, and C=O vibrational modes.

Table S2. The molecular weight distributions.

Sample	Mw	Mn	PDI
PML	7445	2108	3.53
PML-oil	1300	587	2.21
EL	1760	968	1.82
EL-oil	1245	609	2.05
CL	1907	908	2.10
CL-oil	1136	544	2.09

Table S3. Quantification of various lignin bio-oil interunit linkages (%).

Lignin	S/G/H[a]	S/G	*β*-O-4[b]	*β*-*β*[b]	*β*-5[b]	Methoxyl（mmol/g）
CL-oil	30/52/18	0.58	trace	trace	trace	11.3
PML-oil	-99/-	-	trace	trace	trace	12.9
EL-oil	72/28/-	2.57	trace	trace	trace	15.3

[a] Results were expressed per 100 Ar based on quantitative 2D-HSQC spectra. [b]The amount of the specific functional group was expressedas the percentage of *β*–O–4 + *β*–5 + *β*–*β*.

Table S4 Assignment of ^{13}C-^{1}H cross signals in HSQC

Lable	δ_C/δ_H (ppm)	Assignments
OCH_3	55.6/3.7	C-H in methoxyls
A_γ	59.8/3.6	C_γ-H_γ in *β*-O-4 (A)
B_γ	62.1/3.7	C_γ-H_γ in *β*-5 phenylcoumaran (B)
C_γ	70.2/3.5	C_γ-H_γ in *β*-*β* resinol (C)
A_α	71.6/4.85	C_α-H_α in *β*-O-4 substructures linked to a G (A)
A_β	83.2/4.5	C_β-H_β in acylated *β*-O-4 linked to G lignin units (A)
B_α	87.5/5.6	C_α-H_α in *β*-5 phenylcoumaran (B)
$S_{2,6}$	103/6.7	$C_{2,6}$-$H_{2,6}$ in syringil unit (S)
$S'_{2,6}$	107.1/7.3	$C_{2,6}$-$H_{2,6}$, C(α)=O in syringyl units(S')
FA_2	108.6/7.3	C_2-H_2 in ferulic acid (FA)
G_5	113/7.3	C_5-H_5 in guaiacyl unit (G)
G_6	119/6.6	C_6-H_6 in guaiacyl unit (G)
FA_6	122.5/7.1	C_6-H_6 in ferulic acid (FA)
$PCA_{2,6}$	129.8/7.5	$C_{2,6}$-$H_{2,6}$ in *p*-coumalic acid (*p*CA)
$H_{2,6}$	129.1/7.1	$C_{2,6}$-$H_{2,6}$ in *p*-hydroxyphenyl units (H)
PCA_α	144.6/7.5	C_α-H_α in *p*-coumarate (*p*CA)
